# The 5p15.33 Locus Is Associated with Risk of Lung Adenocarcinoma in Never-Smoking Females in Asia

**DOI:** 10.1371/journal.pgen.1001051

**Published:** 2010-08-05

**Authors:** Chao Agnes Hsiung, Qing Lan, Yun-Chul Hong, Chien-Jen Chen, H. Dean Hosgood, I-Shou Chang, Nilanjan Chatterjee, Paul Brennan, Chen Wu, Wei Zheng, Gee-Chen Chang, Tangchun Wu, Jae Yong Park, Chin-Fu Hsiao, Yeul Hong Kim, Hongbing Shen, Adeline Seow, Meredith Yeager, Ying-Huang Tsai, Young Tae Kim, Wong-Ho Chow, Huan Guo, Wen-Chang Wang, Sook Whan Sung, Zhibin Hu, Kuan-Yu Chen, Joo Hyun Kim, Ying Chen, Liming Huang, Kyoung-Mu Lee, Yen-Li Lo, Yu-Tang Gao, Jin Hee Kim, Li Liu, Ming-Shyan Huang, Tae Hoon Jung, Guangfu Jin, Neil Caporaso, Dianke Yu, Chang Ho Kim, Wu-Chou Su, Xiao-Ou Shu, Ping Xu, In-San Kim, Yuh-Min Chen, Hongxia Ma, Min Shen, Sung Ick Cha, Wen Tan, Chin-Hao Chang, Jae Sook Sung, Mingfeng Zhang, Tsung-Ying Yang, Kyong Hwa Park, Jeff Yuenger, Chih-Liang Wang, Jeong-Seon Ryu, Yongbing Xiang, Qifei Deng, Amy Hutchinson, Jun Suk Kim, Qiuyin Cai, Maria Teresa Landi, Chong-Jen Yu, Ju-Yeon Park, Margaret Tucker, Jen-Yu Hung, Chien-Chung Lin, Reury-Perng Perng, Paolo Boffetta, Chih-Yi Chen, Kun-Chieh Chen, Shi-Yi Yang, Chi-Yuan Hu, Chung-Kai Chang, Joseph F. Fraumeni, Stephen Chanock, Pan-Chyr Yang, Nathaniel Rothman, Dongxin Lin

**Affiliations:** 1 Division of Biostatistics and Bioinformatics, Institute of Population Health Sciences, National Health Research Institutes, Zhunan, Taiwan; 2 Division of Cancer Epidemiology, National Cancer Institute, National Institutes of Health, Department of Health and Human Services, Bethesda, Maryland, United States of America; 3 Department of Preventive Medicine, Seoul National University College of Medicine, Seoul, Republic of Korea; 4 Institute of Environmental Medicine, Seoul National University Medical Research Center, Seoul, Republic of Korea; 5 Genomics Research Center, Academia Sinica, Taipei, Taiwan; 6 Graduate Institute of Epidemiology, College of Public Health, National Taiwan University, Taipei, Taiwan; 7 National Institute of Cancer Research, National Health Research Institutes, Zhunan, Taiwan; 8 International Agency for Research on Cancer, Lyon, France; 9 Departments of Etiology and Carcinogenesis and State Key Laboratory of Molecular Oncology, Cancer Institute and Hospital, Chinese Academy of Medical Science and Peking Union Medical College, Beijing, China; 10 Department of Medicine, Vanderbilt Epidemiology Center, Institute for Medicine and Public Health, Vanderbilt-Ingram Cancer Center, Vanderbilt University School of Medicine, Nashville, Tennessee, United States of America; 11 Division of Chest Medicine, Department of Internal Medicine, Taichung Veterans General Hospital, Taichung, Taiwan; 12 Institute of Biomedical Sciences, National Chung-Hsing University, Taichung, Taiwan; 13 School of Medicine, China Medical University, Taichung, Taiwan; 14 Institute of Occupational Medicine and Ministry of Education Key Lab for Environment and Health, School of Public Health, Huazhong University of Science and Technology, Wuhan, China; 15 Department of Internal Medicine, School of Medicine, Kyungpook National University, Daegu, Republic of Korea; 16 Department of Biochemistry, School of Medicine, Kyungpook National University, Daegu, Republic of Korea; 17 Cancer Research Center, Kyungpook National University Hospital, Daegu, Republic of Korea; 18 Genomic Research Center for Lung and Breast/Ovarian Cancers, Korea University Anam Hospital, Seoul, Republic of Korea; 19 Department of Internal Medicine and Division of Brain, Korea University College of Medicine, Seoul, Republic of Korea; 20 Division of Oncology/Hematology, Department of Internal Medicine, Korea University College of Medicine, Seoul, Republic of Korea; 21 Department of Epidemiology and Biostatistics, Cancer Center, Nanjing Medical University, Nanjing, China; 22 Department of Epidemiology and Public Health, National University of Singapore, Singapore, Singapore; 23 Core Genotyping Facility, Advanced Technology Program, Science Applications International Corporation-Frederick, National Cancer Institute, National Institutes of Health, Department of Health and Human Services, Frederick, Maryland, United States of America; 24 Department of Pulmonary and Critical Care, Chang Gung Memorial Hospital, Taiwan; 25 Department of Respiratory Care, Chang Gung University, Taiwan; 26 Cancer Research Institute, Seoul National University College of Medicine, Seoul, Republic of Korea; 27 Department of Thoracic and Cardiovascular Surgery, Clinical Research Institute, Seoul National University Hospital, Seoul, Republic of Korea; 28 Department of Thoracic and Cardiovascular Surgery, Seoul National University College of Medicine, Seoul, Republic of Korea; 29 Department of Thoracic and Cardiovascular Surgery, Seoul National University Bundang Hospital, Geongi-do, Republic of Korea; 30 Department of Internal Medicine, National Taiwan University Hospital and National Taiwan University College of Medicine, Taipei, Taiwan; 31 Department of Epidemiology, Shanghai Cancer Institute, Shanghai, China; 32 Department of Internal Medicine, Kaohsiung Medical University Hospital, Kaohsiung, Taiwan; 33 Department of Internal Medicine, National Cheng Kung University Hospital and College of Medicine, Tainan, Taiwan; 34 Chest Department, Taipei Veterans General Hospital, Taipei, Taiwan; 35 School of Medicine, National Yang-Ming University, Taipei, Taiwan; 36 Department of Internal Medicine, Inha University College of Medicine, Incheon, Republic of Korea; 37 Cancer Center, China Medical University Hospital, Taipei, Taiwan; 38 Laboratory of Translational Genomics, Division of Cancer Epidemiology and Genetics, National Cancer Institute, National Institutes of Health, Department of Health and Human Services, Bethesda, Maryland, United States of America; University of Alabama at Birmingham, United States of America

## Abstract

Genome-wide association studies of lung cancer reported in populations of European background have identified three regions on chromosomes 5p15.33, 6p21.33, and 15q25 that have achieved genome-wide significance with p-values of 10^−7^ or lower. These studies have been performed primarily in cigarette smokers, raising the possibility that the observed associations could be related to tobacco use, lung carcinogenesis, or both. Since most women in Asia do not smoke, we conducted a genome-wide association study of lung adenocarcinoma in never-smoking females (584 cases, 585 controls) among Han Chinese in Taiwan and found that the most significant association was for rs2736100 on chromosome 5p15.33 (p = 1.30×10^−11^). This finding was independently replicated in seven studies from East Asia totaling 1,164 lung adenocarcinomas and 1,736 controls (p = 5.38×10^−11^). A pooled analysis achieved genome-wide significance for rs2736100. This SNP marker localizes to the *CLPTM1L*-*TERT* locus on chromosome 5p15.33 (p = 2.60×10^−20^, allelic risk = 1.54, 95% Confidence Interval (CI) 1.41–1.68). Risks for heterozygote and homozygote carriers of the minor allele were 1.62 (95% CI; 1.40–1.87), and 2.35 (95% CI: 1.95–2.83), respectively. In summary, our results show that genetic variation in the *CLPTM1L-TERT* locus of chromosome 5p15.33 is directly associated with the risk of lung cancer, most notably adenocarcinoma.

## Author Summary

Worldwide, approximately 15% of lung cancer cases occur among nonsmokers. Genome-wide association studies (GWAS) of lung cancer conducted in populations of European background have identified three regions on chromosomes 5, 6, and 15 that harbor genetic variants that confer risk for lung cancer. Prior studies were conducted primarily in cigarette smokers, raising the possibility that the associations could be related to tobacco use, lung carcinogenesis, or both. A GWAS of lung cancer among never-smokers is an optimal setting to discover effects that are independent of smoking. Since most women in Asia do not smoke, we conducted a GWAS of lung adenocarcinoma among never-smoking females (584 cases, 585 controls) in Taiwan, and observed a region on chromosome 5 significantly associated with risk for lung cancer in never-smoking women. The finding was independently replicated in seven studies from East Asia totaling 1,164 lung adenocarcinomas and 1,736 controls. To our knowledge, this study is the first reported GWAS of lung cancer in East Asian women, and together with the replication studies represents the largest genetic association study in this population. The findings provide insight into the genetic contribution of common variants to lung carcinogenesis.

## Introduction

To date, several large genome-wide association studies (GWAS) of lung cancer conducted in subjects of European background have identified susceptibility alleles on chromosomes 5p15.33, 6p21.33 and 15q25 [1]–[8]. These studies have shown that statistical evidence that exceeds the threshold of genome wide significance, defined as a p value less than 5×10^−7^ [9] or 1×10^−8^ [10]. In each study, the majority of cases and controls were cigarette smokers, making it difficult to determine whether these loci are associated with lung carcinogenesis or tobacco use, or perhaps both [11]. It has been difficult to accrue a sufficiently large set of lung cancer cases with no history of smoking because a high proportion of lung cancer in women as well as men in North America and Europe is directly related to tobacco use. In contrast, a substantial proportion of lung cancer in East Asian women occurs among non-smokers, who interestingly have a relatively high rate of lung cancer [12]. This suggests that genetic and/or environmental factors could account for the observed differences. To investigate this further, we conducted a genome-wide association study with follow-up of notable SNPs in never-smoking women in East Asia. In addition, we genotyped tag SNPs optimized for East Asians for the three regions previously identified by GWAS in European populations.

## Results

### Genome-wide association scan

**Figure 1:**
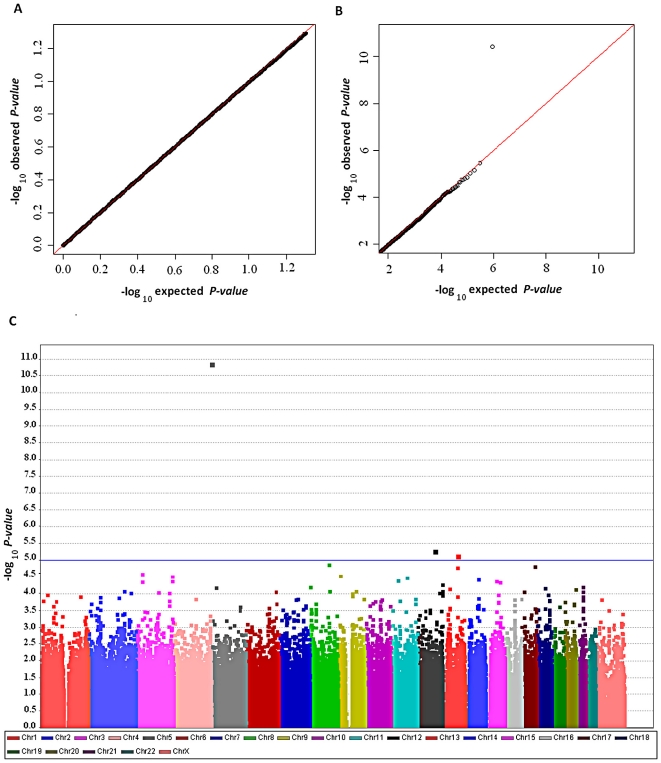
Genome-wide association results in the GELAC study. (A) Quantile-quantile plot for lower 90% of −log *P*-values, (B) upper 10% of −log *P*-values, and (C) scatter plot of *P*-values in−log scale from the trend test for 457,504 genotyped variants comparing 584 cases and 585 controls.

We conducted an initial GWAS of 584 lung cancer cases and 585 controls drawn from a case-control study in Taiwan, the Genetic Epidemiological Study of Lung Adenocarcinoma (GELAC) [13] (Table 1). Cases were restricted to those never-smoking females with a confirmed diagnosis of adenocarcinoma of the lung in GELAC. Controls were drawn from never-smoking female controls in GELAC and frequency-matched by age with cases (see Text S1 for more details). We began with a pilot study in which 54 cases and 54 controls were genotyped with the Illumina HumanCNV370-Duo BeadChip and, based on its success, 550 cases and 549 controls were genotyped on the Illumina HumanHap 610 Quad BeadChip. After quality control metrics were applied to both data sets (see Materials and Methods), the variance inflation factor λ in the genomic control model was found to be 1.013 and the inflation factor λ_1000_ for an equivalent study of 1000 cases and controls [14] was 1.022; together with the comparison of the observed and expected p-values in the quantile-quantile plot, shown in Figure 1, there is no evidence of a substantial issue related to population substructure but instead, several promising regions in the tail of the distribution are apparent, suitable for follow-up analysis. In fact, the distribution of the bottom 90% of p-values is similar to the expected distribution (Figure 1A) whereas the top 10% p-values displayed a deviation consistent with possible new signals (Figure 1B).

**Table 1:** Number of cases and controls, and study characteristics, for each participating study center.

		Cases	Controls	Age [mean (std)]		
Study Center†	Region	(n = 2768)	(n = 3100)	Controls	cases	Study Design
GELAC	Taiwan (GWAS)	584	585	58.9 (11.2)	59.0 (11.4)	Case-control
GELAC	Taiwan (replication)	610	560	57.0 (13.8)	60.1 (11.6)	Case-control
CAMSCH	Mainland China	287	287	56.3 (11.6)	56.3 (10.0)	Case-control
SNU	South Korea	259	293	61.2 (10.2)	61.2 (10.5)	Case-control
SWHS	Mainland China	209	213	58.9 (8.4)	58.9 (8.3)	Cohort
WHLCS	Mainland China	207	207	56.9 (9.6)	56.3 (11.2)	Case-control
KNUH	South Korea	121	119	60.7 (6.5)	61.1 (9.4)	Case-control
KUMC	South Korea	95	87	60.9 (6.4)	62.7 (10.5)	Case-control
GEL-S*	Singapore	193	546	64.7 (11.4)	63.4 (12.1)	Case-control
NJLCS*	Mainland China	203	203	56.9 (9.8)	56.8 (9.7)	Case-control

**†:** GELAC: Genetic Epidemiological Study of Lung Adenocarcinoma (in Taiwan); CAMSCH: Chinese Academy of Medical Sciences Cancer Hospital Study; SNU: Seoul National University study; SWHS: Shanghai Women's Health Cohort Study; WHLCS: Wuhan Lung Cancer Study; KNUH: Kyungpook National University Study; KUMC: Korea University Study; GEL-S: Genes and Environment in Lung Cancer, Singapore study; NJLCS: Nanjing Lung Cancer Study.

*A subset of data presented in this paper for CAMSCH (Wu et al., (2009) Cancer Res;12:5065–5072), GEL-S (Truong et al., (2010) J Natl Cancer Inst, In press ), and NJLCS (Jin et al., (2009) Carcinogenesis;30:987–990;Wu et al., (2009) Cancer Res;12:5065–5072) was previously published and is noted in Tables S1, S2, S4, S6 and Figure S1.

**Figure 2:**
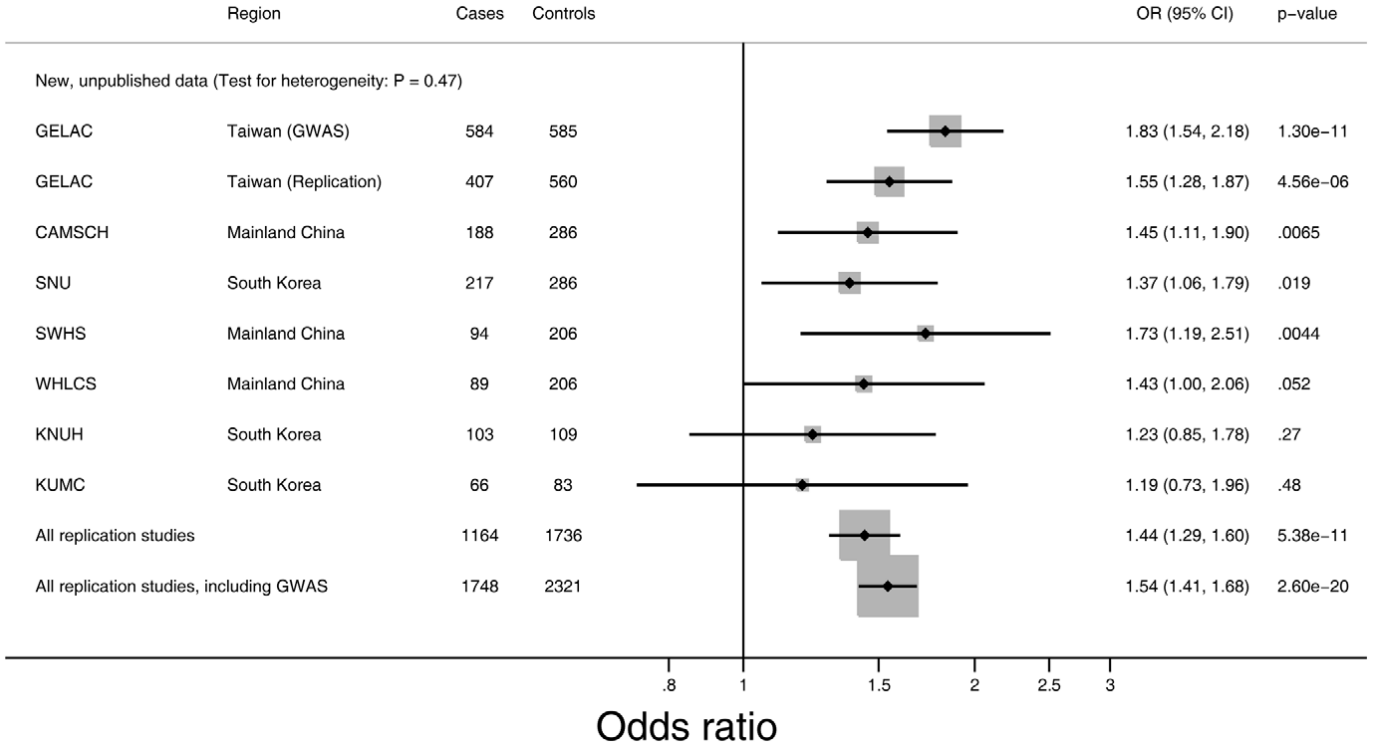
Risk of lung adenocarcinoma associated with rs2736100 for never-smoking female cases and never-smoking female controls from East Asia. Forest plot representing lung adenocarcinoma risk and the rs2736100 genotype. Odds ratios (OR) and 95% confidence intervals (CI) for lung adenocarcinoma are derived from the per-allele model. All models are adjusted for age and study center. GELAC: Genetic Epidemiological Study of Lung Adenocarcinoma (in Taiwan); CAMSCH: Chinese Academy of Medical Sciences Cancer Hospital Study; SNU: Seoul National University study; SWHS: Shanghai Women's Health Cohort Study; WHLCS: Wuhan Lung Cancer Study; KNUH: Kyungpook National University Study; KUMC: Korea University Study.

As shown in Figure 1C, the scatter plot of p-values on a −log scale for the trend test conducted for 457,504 SNPs used after quality control metrics were applied, only one SNP, rs2736100, was highly associated with lung cancer (p = 1.30 * 10^−11^) below the threshold of genome-wide significance, namely, p less than 1×10^−7^ (Figure 2). In an analysis for trend adjusted for age, the allelic odds ratio was 1.83 (1.54–2.18), which is notably higher than the estimates reported in the European studies (Figure 2) [4]. It is remarkable that our finding suggests a higher estimated effect size compared to that which was reported in Europeans, who were primarily smokers. To confirm the signal at rs2736100, the samples in the GWAS were genotyped using an optimized TaqMan assay (ABI, Foster City, CA), which had a concordance of 99.7% between the two platforms [15].

### Replication of the association of rs2736100 with lung cancer risk

Replication of the strongest signal, rs2736100 was performed in the remaining subjects of the GELAC study [13] as well as six studies of never-smoking Asian women with lung adenocarcinoma in East Asia. A total of 1164 cases with lung adenocarcinoma and 1736 controls were genotyped using an optimized TaqMan assay (shown to have high concordance with the Illumina results as described above). The additional replication studies included the Chinese Academy of Medical Sciences Cancer Hospital study (CAMSCH) [16], Wuhan lung cancer study (WHLCS) [17], Seoul National University study (SNU) [18], Korea University Medical Center study (KUMC) [19], Kyungpook National University Hospital study (KNUH) [20] and Shanghai Women's Health Cohort Study (SWHS) [21], [22] (Table 1). Characteristics of the study subjects from the GWAS and the replication studies are presented in Table 1.

The combined replication study confirmed that rs2736100 is associated with risk for lung adenocarcinoma in never-smoking women in East Asia (p = 5.38 * 10^−11^; allelic OR = 1.44; 95% CI 1.29–1.60) (Figure 2). In a pooled analysis of the GWAS and replication studies, rs2736100 was conclusively associated with the risk for lung adenocarcinoma in never-smoking females in East Asian populations; the allelic OR is 1.54 (95% CI 1.41–1.68; p = 2.60 * 10^−20^) (Figure 2 and Table 2). The estimated odds ratios for the heterozygous and homozygous carriers are 1.62 (95% CI 1.40–1.87) and 2.35 (95% CI 1.95–2.83). There was no evidence of heterogeneity between the results of the one cohort study (SWHS) and the pooled analysis of the 6 case-control studies (p = 0.36). Further pooling with two previously published studies, the Nanjing lung cancer study (NJLCS) [23] and the Genes and Environment in Lung Cancer, Singapore study (GEL-S) [24], [25] (Table 1, Table S1), yielded comparable results (p = 1.16 * 10^−21^) (Table S2, Figure S1). Across all studies, we observed consistently increased risk associated with rs2736100 with no evidence for heterogeneity between studies, measured by the I^2^ test for heterogeneity (Figure S1). In a subsequent analysis combining all lung cancer cases, rs2736100 was also significantly associated with lung cancer susceptibility (p = 5.50 * 10^−20^; allelic OR = 1.48; 95% CI 1.36–1.62) (Table 2). This observation is comparable to what is being reported for adenocarcinoma alone, which is not surprising because adenocarcinomas constitute 76% of cases (Table 2).

**Table 2:** Lung cancer risk associated with rs2736100, among never-smoking females from East Asia, by histology.

Genotype	Controls	%	Cases	%	OR‡	95%CI‡	p-value
	**all lung cancers**						
TT	852	36.7	599	26.0			
GT	1132	48.8	1187	51.4	1.49	1.30–1.70	3.76E-09
GG	337	14.5	522	22.6	2.20	1.85–2.62	2.94E-19
trend	2321		2308		1.48	1.36–1.62	5.50E-20
	**adenocarcinomas**						
TT	852	36.7	428	24.5			
GT	1132	48.8	922	52.7	1.62	1.40–1.87	8.51E-11
GG	337	14.5	398	22.8	2.35	1.95–2.83	3.05E-19
trend	2321		1748		1.54	1.41–1.68	2.60E-20
	**squamous cell carcinomas**						
TT	852	36.7	60	33.9			
GT	1132	48.8	82	46.3	1.02	0.72–1.44	0.91
GG	337	14.5	35	19.8	1.47	0.95–2.27	0.084
trend	2321		177		1.18	0.95–1.48	0.14

**‡:** Odds ratios (OR) and 95% confidence intervals (CI) adjusted for age and study center.

**Figure 3:**
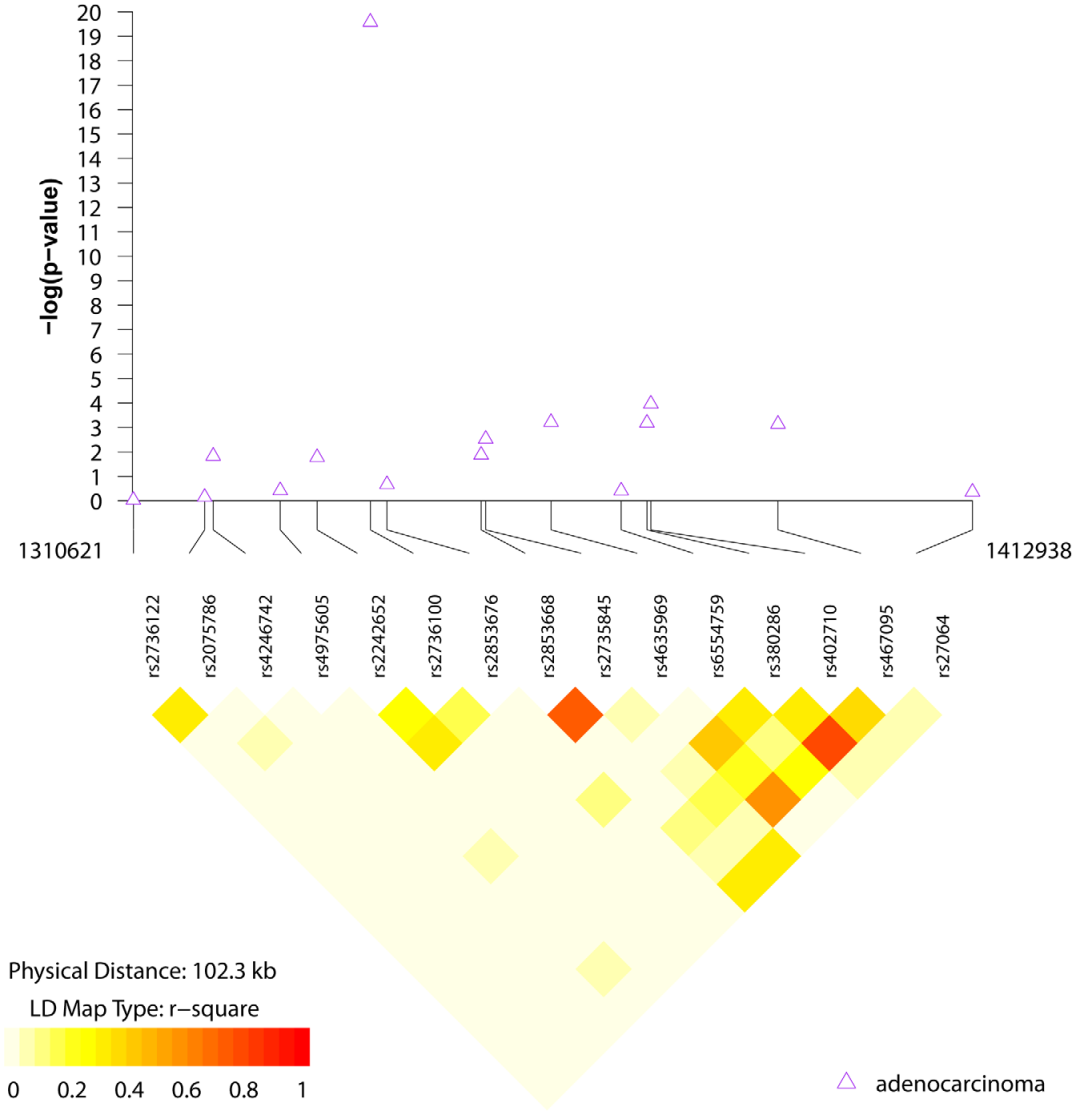
LD structure and association results for the chr5p15 region and lung adenocarcinoma. LD structure and regional association results for 15 SNPs genotyped in the 5p15 region. SNPs included were tagged with *r*_2_≥0.8 in HapMap CHB [37].

We conducted a first generation fine mapping of this region of chromosome 5p15.33 using 15 tag SNPs optimized in the East Asian studies in HapMap phase 2; the 15 SNPs were chosen using an r^2^≥0.8 as a threshold and estimated to cover approximately 85% of the known SNPs in HapMap phase 2 (Table S3). We did not identify stronger signals for association with lung adenocarcinoma in the 15 SNPs, as shown in Figure 3 and Table S4. Notably, rs402710, previously reported in GWAS of European ancestry [4], was the second most significant SNP tested in this region but did not achieve genome-wide significance (p = 0.0046) (Table S4). When rs402710 and rs2736100 were analyzed in a multivariable model, the former became non-significant (p = 0.33).

## Discussion

In this study of lung adenocarcinoma in East Asian never-smoking women, we report a highly significant association with the common SNP, rs2736100, which localizes to the *TERT-CLPTM1L* locus on chromosome 5p15.33. Our study is notable because the sample size for never-smoking female cases is substantially larger than previous reports. Moreover, the estimated effect size observed for rs2736100 and adenocarcinoma of the lung (OR = 1.54) is greater than the associations previously reported in European populations (e.g., OR = 1.24 from the largest meta-analysis reported to date [4], [8], p = 0.000046 for difference). Our study provides strong evidence that this locus on chromosome 5p15.33 is directly related to lung carcinogenesis because it has been conclusively shown in non-smoking women.

The SNP marker, rs2736100, is mapped to a region of chromosome 5p15.33 in which common and rare genetic variants have been linked to a spectrum of cancers and related conditions. rs2736100 is localized to intron 2 of the telomerase gene *TERT*, a reverse transcriptase that is critical for telomere replication and stabilization by controlling telomere length. Variants in the *TERT-CLPTM1L* locus have been identified by GWAS to harbor susceptibility alleles for cancer of the brain, pancreas and lung [8], [26], [27]. For the latter, a large meta-analysis combined with a new scan indicates that the signal in this locus is most strongly associated with one histology, adenocarcinoma in studies of European subjects [8].

There is further evidence for association of this locus with additional cancers, though the reported results have not yet achieved the genome-wide association threshold; these include cancer of the bladder, prostate, uterine cervix, and skin including basal cell carcinoma and melanoma [4], [5], [7], [27]. Rare variations/mutations in the *TERT* gene have been described as a risk factor for acute myelogenous leukemia and also explain a proportion of the inherited bone marrow failure family pedigrees with dyskeratosis congenita, a cancer predisposition syndrome [28], [29]. Mutations in the *TERT* gene have also been described in patients with idiopathic pulmonary fibrosis [30], [31]. Together these findings suggest that the *TERT-CLPTM1L* 5p15.33 region could be important in the development of a spectrum of cancers. Still, at this time, further studies are needed to fine map the region, based on comprehensive re-sequence analysis in East Asian populations, to narrow the set of genetic variants worthy of functional studies to establish the mechanism underpinning the association marked by the SNP rs2736100 and subsequently compare these findings with comparable analyses in the other diseases.

The plausible mechanisms underlying the association signals across this region of chromosome 5p15.33 are currently under active investigation by many groups. Our findings are particularly interesting because we have identified variants that appear to be directly related to primary carcinogenesis. In this regard, it is critical that future studies evaluate environmental risk factors that may contribute, particularly since there is preliminary data suggesting that smoking as well as other exposures could directly influence telomere lengths [32]. It is noteworthy that lung cancer risk among non-smoking women in East Asia has been linked to indoor air pollution from environmental tobacco smoke [12], fumes produced by high temperature cooking [33], and coal combustion products [34].

Based on the discovery of susceptibility loci on chromosomal regions 6p21.33 and 15q25 first observed in European populations [1]–[3], [35], we attempted to replicate the findings in never-smoking women in East Asia. The strongest SNPs reported in each region plus additional tag SNPs, chosen on the basis of HapMap Phase 2, were genotyped in seven studies. 15 SNPs were selected for 6p21.33, covering an estimated 93% of known SNPs in HapMap phase 2 in the East Asian populations, whereas 24 SNPs were genotyped across 15q25, covering an estimated 83% of known common SNPs in the region (Table S3). In these East Asian never-smoking women, there was no convincing evidence for association at chromosome 6p21.33 or for 15q25 for lung cancer overall or for the adenocarcinoma subtype (Tables S5 and S6).

We report conclusive evidence that common genetic variants in the *TERT-CLPTM1L* locus on chromosome 5p15.33 are associated with risk for lung adenocarcinoma in non-smoking Asian women. We observed estimated effect sizes that are substantially higher than those previously reported in European smokers, which bears follow-up investigation into the biology of the underlying mechanism of the contribution of this region to primary lung carcinogenesis. Since this region on chromosome 5p15.33 has been implicated in many cancers, our observations should stimulate further investigation of the region that could lead to new insights into carcinogenesis.

## Materials and Methods

### Studies

A description of each study is provided in Table 1 and Text S1. Lung cancer cases and controls for the GWAS were drawn from the Genetic Epidemiological Study of Lung Adenocarcinoma (GELAC) in Taiwan. A total of 584 never-smoking incident cases and 585 never-smoking controls were included in the GWAS. The replication studies were drawn from seven studies, including additional subjects from the GELAC study [13], the Chinese Academy of Medical Sciences Cancer Hospital study (CAMSCH) [16], the Wuhan lung cancer study (WHLCS) [17], the Seoul National University study (SNU) [18], the Korea University Medical Center study (KUMC) [19], the Kyungpook National University Hospital study (KNUH) [20], and the Shanghai Women's Health Cohort Study (SWHS) [21], [22]. In addition, data were pooled with previously published findings from the Nanjing lung cancer study (NJLCS) [23] and the Genes and Environment in Lung Cancer, Singapore study (GEL-S) [24] (Table 1). All studies are case-control studies with the exception of the SWHS, which is a prospective cohort study. The range of ages is similar in cases and controls across all studies (Table 1).

### Ethics statement

All study subjects provided informed consent and each study was approved by its respective institution's IRB.

### Genotyping and quality control

#### Genome-wide association study genotyping and quality control

GWAS genotyping of the GELAC samples was performed in two separate phases. In the pilot phase, 54 cases and 54 controls were genotyped by GeneTech Biotech Co., (Taiwan), using the Illumina HumanCNV370-Duo BeadChip. The cases were never-smoking females diagnosed with lung adenocarcinoma at age ≤51 who had questionnaire data and DNA that passed quality control criteria for scanning. The controls were never-smoking females matched by age (±2 years) to cases.

Cluster definitions were determined using Illumina BeadStudio Genotyping Module v.3.3.4. Genotype calls were based on a quality score (Gene call value) of 0.25 or higher. Four blind duplicate pairs were included, and the concordance of SNP genotype calls between each pair is greater than 99.997%. Quality control metrics for data from the first phase are similar to those for data from the second phase, detailed below.

In the second phase of the GWAS, 550 cases and 549 controls were genotyped with the Illumina HumanHap610 Quad BeadChip on contract at deCODE Genetics, Iceland. The cases were the first never-smoking female lung adenocarcinoma subjects to be enrolled in the study with questionnaire data and DNA that passed quality control for scanning. Cluster definitions were determined using the Illumina BeadStudio Genotyping Module. The median genotype call rate for samples was 99.78%. 95% overall displayed call rates larger than 99.49%; the median call rate for variants is 99.91%, with 95% of variants with call rates above 99.55%. 21 blind duplicate pairs displayed an average concordance greater than 99.99%.

After quality control metrics were applied, 457,504 SNPs were used for the association analysis. SNPs (n = 1,705) were excluded if the call rate was below 90%, (i.e., a missing rate larger than 0.1); SNPs with a minor allele frequency below 0.05 (n = 131,558); SNPs with missing rate between 0.02 and 0.1 and non-random genotype failure with p<0.02 (n = 1,046); and, significant deviation from fitness for Hardy-Weinberg equilibrium (p<0.0001 in controls) (n = 718). 1064 unique samples from phase 2 were used in the association analysis, after two exclusion steps. The first set of exclusions was based on the quality control metrics described above and relatedness among individuals: call rates less than 90% (n = 3); sex discrepancies based on the X chromosome heterozygosity (n = 7); contaminated samples with high heterozygosity scores (n = 4), first or second degree relatives identified using genome-wide pairwise identical by descent (IBD) estimates (n = 9).

We further excluded 12 individuals from phase 2, based on population substructure analysis. In fact, to detect differences in population substructure, pairwise population concordance (PPC) test in PLINK (http://pngu.mgh.harvard.edu/purcell/plink/) [36] were performed with a threshold of 10^−20^ on two data sets using all autosomal SNPs that had passed the quality control metrics described above. The first data set consists of the 1184 unrelated individuals with high quality genotype data (108 from phase 1 and 1076 from phase 2). The PPC test identified 15 outliers who were distinct from the remaining 1169 (105 from phase 1 and 1064 from phase 2). The eight self-described aborigines (2 in Phase 1 and 6 in Phase 2) were among the outliers. Based on the PPC analysis, the final genome-wide association analysis was conducted using 1169 samples.

To further assess the population homogeneity in our study sample, we conducted additional analyses in our 1184 individuals with HapMap3 release 2 data [37]. The results indicate that for the 1184 unrelated individuals with high quality genotype data, 15 outliers were detected, thus yielding 1169 individuals with homogeneous genetic structure available for follow-up analyses. We seeded the study population with genotype data from hapmap 3 as well as hapmap 2; this included 85 CHD (Chinese in Metropolitan Denver, Colorado), in addition to our 1184 individuals and the hapmap 2 (84 CHB (Han Chinese in Beijing, China) and 86 JPT (Japanese in Tokyo, Japan)). A second analysis included our 1184 study individuals and a larger sample of HapMap3 release 2, namely the CHB, CHD, GIH (Gujarati Indians in Houston, Texas), JPT, LWK (Luhya in Webuye, Kenya), MKK (Maasai in Kinyawa, Kenya), and TSI (Toscani in Italia): the results confirmed that 15 outliers were detected whereas the 1164 represented a homogeneous population.

Although the above PPC tests seem to suggest little population substructure in our 1169 samples, we still used EIGENSTRAT [38] to conduct GWAS analysis to correct possible population stratification. We found that for the SNP rs2736100, the P-value is 1.239×10^−11^ based on the Armitage trend Chi-square statistic with no stratification correction and the P-value is 2.764×10^−11^ based on EIGENSTRAT using 10 principal components (the default value) for stratification correction. There was a negligible difference in p-values with and without this correction.

The genotyping cluster plot generated by the Illumina platform for rs2736100 is presented in Figure S2. The adjusted intensities for each allele are plotted, where each color represents a different genotype in the cluster plots. As shown in the figure, clusters of different genotypes are well separated from each other, indicating a high confidence in genotype calling in our study. The genotype call at this locus was confirmed with TaqMan genotyping (concordance of 99.7%).

#### Replication SNP selection and genotyping

DNA was extracted from blood samples and genotyped at the National Cancer Institute Core Genotyping Facility (CGF) (Http://cgf.nci.nih.gov) for four studies, SNU, KUMC, KNUH, and SWHS. TaqMan genotyping for the GELAC study (including all previously scanned cases and controls plus remaining never-smoking female cases and their matched controls) and the GEL-S studies was conducted in Taiwan and Singapore, respectively. Genotyping for the CAMSCH, WHLCS, and NJLCS studies were conducted at the Cancer Institute and Hospital, Chinese Academy of Medical Science, using TaqMan assays designed and optimized by the CGF (http://snp500cancer.nci.nih.gov).

We selected 54 SNPs optimized for Eastern Asian populations to cover the three chromosomal regions previously reported to show association for lung cancer (i.e., 15 SNPs in 5p15, 15 SNPs in 6p, and 24 SNPs in 15q25) (Table S3). The coordinates for selecting the tag SNPs were based on an r^2^<0.8 using the CHB samples of HapMap phase 2. The boundaries for the tag SNP selection were as follows: 5p15.33 from 1310620 to 1412939, 6p21.33 from 28782776 to 29018856 and 15q25 from 76593077 to 76702301 (Build 37). We computed genomic coverage using the GLU software package (http://code.google.com/p/glu-genetics/) for common SNPs (MAF≥0.05) based on the most recent build (Build 37) of the HapMap CHB [37] genotype data.

All TaqMan assays (Applied Biosystems Inc., Foster City, CA) for this study were optimized on the ABI 7900HT detection system with high concordance with sequence analysis of 102 individuals as listed on the SNP500Cancer website (http://snp500cancer.nci.nih.gov). All of the genotype frequencies were consistent with Hardy-Weinberg equilibrium except three SNPs (rs402710, rs9368570, and rs9257280) using a chi-square test (*P*<0.0001, Table S3). All reported genotyped results are based on completion rates of greater than 94% across all studies.

### Statistical analysis

#### Genome-wide association tests

The program PLINK [36] was used to conduct primary statistical tests for association in the discovery phase. Association analyses between individual SNP and the lung cancer risk were carried out using computer packages in PLINK. Q-Q plots analyzed by the trend test are shown in Figure 1. We note that for Phase 1, we imputed the genotypes at the SNPs contained in HumanHap 610 Quad BeadChip but not in HumanCNV370-Duo BeadChip by using IMPUTE developed by Marchini et al. [39] and haplotypes of CHB in HapMap as the reference.

#### Replication and pooled analyses

Unconditional logistic regression was used to estimate the ORs and 95% CIs, adjusting for age and study center. All p values are two-sided. The most prevalent homozygous genotype was used as the reference group. Tests for trend were conducted by assigning the ordinal values 1, 2, and 3 to the most prevalent genotypes in rank order of wild type, heterozygous, and variant homozygous genotypes, respectively.

## Supporting Information

Figure S1 Risk of lung cancer associated with rs2736100 for never-smoking female adenocarcinoma cases and never-smoking female controls from East Asia.
(0.66 MB TIF)

Figure S2 SNP graph of rs2736100 from (A) Illumina 610K (B) Illumina 370K based on Beadstudio Genotyping Module v3.
(1.47 MB TIF)

Table S1 Lung cancer risk associated with rs2736100 among never-smoking females from East Asia, by study center, including two previously published studies.
(0.04 MB XLS)

Table S2 Lung cancer risk associated with rs2736100 among never-smoking females from East Asia, by histology, including two previously published studies.
(0.03 MB XLS)

Table S3 Chromosome 5, 6, and 15 SNPs genotyped.
(0.04 MB XLS)

Table S4 Lung cancer risk associated with chromosome 5 SNPs, among never-smoking females from East Asia.
(0.05 MB XLS)

Table S5 Lung cancer risk associated with chromosome 6 SNPs, among never-smoking females from East Asia.
(0.07 MB XLS)

Table S6 Lung cancer risk associated with chromosome 15 SNPs, among never-smoking females from East Asia.
(0.07 MB XLS)

Text S1 Supplementary information.
(0.09 MB DOC)
