# Supplementary figures and images for: The 5p15.33 Locus Is Associated with Risk of Lung Adenocarcinoma in Never-Smoking Females in Asia
Source: PLoS Genet. 2010 Aug 5;6(8):e1001051. doi: 10.1371/journal.pgen.1001051 (PMC2916850; doi:10.1371/journal.pgen.1001051)

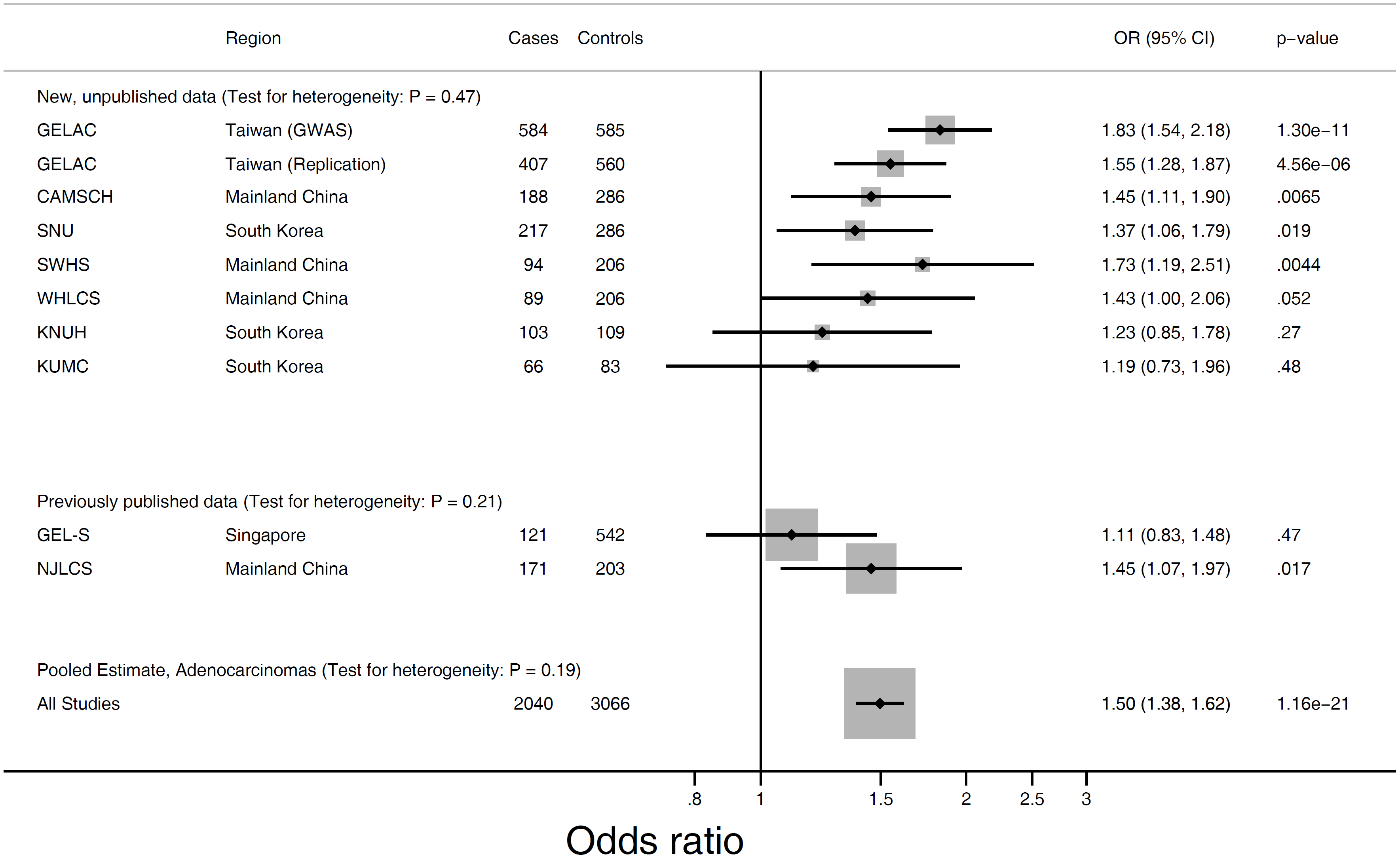

Supplement: Figure S1 — Risk of lung cancer associated with rs2736100 for never-smoking female adenocarcinoma cases and never-smoking female controls from East Asia. (0.66 MB TIF) [file pgen.1001051.s001.tif]

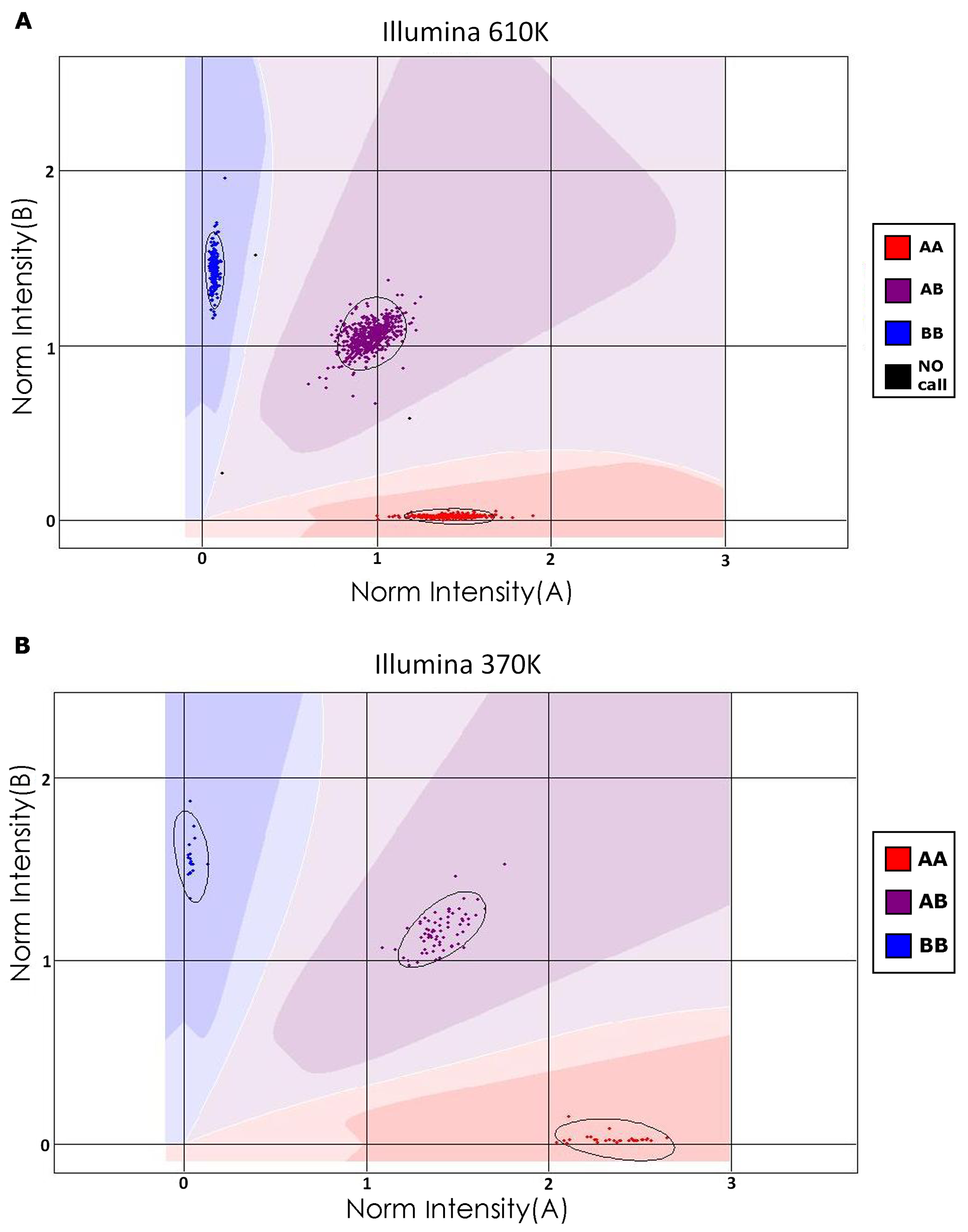

Supplement: Figure S2 — SNP graph of rs2736100 from (A) Illumina 610K (B) Illumina 370K based on Beadstudio Genotyping Module v3. (1.47 MB TIF) [file pgen.1001051.s002.tif]
