# Supplementary material for: The 5p15.33 Locus Is Associated with Risk of Lung Adenocarcinoma in Never-Smoking Females in Asia
Source: PLoS Genet. 2010 Aug 5;6(8):e1001051. doi: 10.1371/journal.pgen.1001051 (PMC2916850; doi:10.1371/journal.pgen.1001051)
Supplement: Text S1 — Supplementary information. (0.09 MB DOC) [file pgen.1001051.s009.doc]

**Supplementary Information**

**Supplementary Methods**

**Study populations.** The baseline characteristics of the subjects are shown in Table 1. Studies included the Genetic Epidemiological Study of Lung Adenocarcinoma (GELAC) study [1], the Chinese Academy of Medical Sciences Cancer Hospital Study (CAMSCH) [2], the Wuhan lung cancer study (WHLCS) [3], the Seoul National University study (SNU) [4], the Korea University Medical Center study (KUMC) [5], the Kyungpook National University Hospital study (KNUH) [6], the Shanghai Women’s Health Cohort Study (SWHS) [7,8], the Nanjing lung cancer study (NJLCS) [9], and the Genes and Environment in Lung Cancer, Singapore study (GEL-S) [10] (Table 1). The GELAC, CAMSCH, SNU, KNUH, KUMC, GEL-S, and NJLCS are population-based case-control studies; the WHLCS is a hospital-based case-control study; and the SWHS is a prospective cohort study. Age was similar among cases and controls for these studies (Table 1).

All study subjects provided informed consent and each study was approved by its respective institution’s IRB.

In the GELAC study, cases were recruited between January 2002 and December 2009 from six hospitals, National Taiwan University Hospital, Taipei Veterans General Hospital, Chang-Gung Memorial Hospital, Taichung Veterans General Hospital, National Cheng-Kung University Hospital, and Kaohsiung Medical University Hospital in Taiwan. For the study, cases were 18 years or more of age with incident primary lung cancer. Control subjects were cancer-free, randomly selected from the health examination clinics of the same hospitals during the same time period of case recruitment and frequency matched by age, gender. The study is described in [1]. All study subjects were ethnic Chinese.

The CAMSCH study is described in [2]. The study was carried out between 1997 and 2008. All of the recruited subjects were ethnic Han Chinese. Cases were recruited from Beijing city and from surrounding provinces at the Chinese Academy of Medical Sciences (Beijing, China) Cancer Hospital. Controls were from a community nutritional survey. Subjects were cancer-free individuals in the same region during the same period as the cases were collected.

The WHLCS study was carried out between July 2002 and November 2004 and is described in [3]. All of the subjects were ethnic Han Chinese. Cases were consecutively recruited from the Wuhan Zhongnan Hospital. There were no restrictions based on age, sex, and histology, but patients with a previous cancer history or with unknown conditions of radiotherapy or chemotherapy were excluded. Controls were cancer-free outpatients from other clinics in the same hospital during the same period when the cases were recruited. The study has been described elsewhere [3]. All controls were frequency matched to the cases by age, sex, and residential area (urban or countryside).

The SNU study of lung cancer in Korea was conducted between 2001 and 2008 and is described in [4]. The lung cancer patients were recruited from patients at Seoul National University Hospitals in Seoul and Bundang, while the age- and sex-matched controls were participants in the health checkup program of Inha University Hospital. Cases were recruited from Seoul National University Hospitals in Seoul and Bundang between 2005 and 2007, and between 2001 and 2008, respectively with no age, histological or stage restrictions. The controls were without lung cancer admitted to the health checkup program of Inha University Hospital between 2001 and 2004 were recruited. Eligibility criteria only included subjects without any history of lung cancer. All study subjects were ethnic Koreans.

The KUMC study was carried out between August 2001 and February 2008 and is described in [5]. Lung cancer patients were recruited from the patient pool at the Genomic Research Center for Lung and Breast/Ovarian Cancer and the Inha University Medical Center. Age- and sex-matched controls were participants in the health checkup program of Inha University Hospital. All study subjects were ethnic Koreans.

The KNUH study was conducted between January 2006 and December 2007 and is described in [6]. Cases were recruited from the patient pool at the Kyungpook National University Hospital. The control subjects were randomly selected from a pool of healthy volunteers who visited the general health check-up center at the Hospital. All study subjects were ethnic Koreans.

The SHWS is described in [7,8]. Between 1996 and 2000, a total of 75,221 Chinese women from urban Shanghai, China, ages 40 to 70 years, participated in the study and completed the baseline surveys. Of those, 279 women who were found to be younger than age 40 years or older than age 70 years, 1,490 women who had a prevalent case of cancer, and 10 women who did not accrue any follow-up time, were excluded. The remaining women were followed through December 2008. Three biennial in-person follow-ups for all living cohort members were conducted by in-home visits between 2000 and 2002, 2002 and 2004, and 2004 and 2006 with response rates of 99.8, 98.7 and 94.9%, respectively. A nested case-control study design was used in this cohort. Incident cases diagnosed between 1996 and December 2008 with malignant neoplasm of the bronchus or lung were included in this study. Controls were selected among the study participants in the cohort who were cancer free at the time of cancer diagnosis of the matched cases. One control was randomly selected and matched with each case by age at baseline (±2 years). In total, 209 case and 213 control subjects were selected for the present study.

The NJLCS was conducted between 2002 and 2008 and is described in [9]. Study subjects were Han Chinese. The cases were recruited at the Cancer Hospital of Jiangsu Province, the First Affiliated Hospital of Nanjing Medical University, and the Nanjing Thoracic Hospital (Nanjing, China). Controls were cancer-free individuals who participated in a community-based screening program for noninfectious diseases, and were randomly selected from a pool of 30,000 in Jiangsu Province. They were frequency-matched with cases based on age and sex.

The GEL-S study is a hospital-based case-control study from February 2005 to January 2008 in the 5 major public-sector hospitals in Singapore and is described in [10]. Eligible cases were Chinese women with a diagnosis of primary lung carcinoma (all histological types). Controls were selected from the same hospital, frequency-matched by 10-year age groups and admitted or seen in the hospital within 30 days of the date of diagnosis of the corresponding case. Exclusion criteria were admissions for the diagnosis or management of malignancy or chronic respiratory disease (excluding tuberculosis).

Reference List

1. Jou YS, Lo YL, Hsiao CF, Chang GC, Tsai YH et al. (2009) Association of an EGFR intron 1 SNP with never-smoking female lung adenocarcinoma patients. Lung Cancer 64: 251-256.

2. Wu C, Hu Z, Yu D, Huang L, Jin G et al. (2009) Genetic variants on chromosome 15q25 associated with lung cancer risk in Chinese populations. Cancer Res 69: 5065-5072.

3. Bai Y, Xu L, Yang X, Hu Z, Yuan J et al. (2007) Sequence variations in DNA repair gene XPC is associated with lung cancer risk in a Chinese population: a case-control study. BMC Cancer 7: 81.

4. Kim JH, Kim H, Lee KY, Choe KH, Ryu JS et al. (2006) Genetic polymorphisms of ataxia telangiectasia mutated affect lung cancer risk. Hum Mol Genet 15: 1181-1186.

5. Jung HY, Whang YM, Sung JS, Shin HD, Park BL et al. (2008) Association study of TP53 polymorphisms with lung cancer in a Korean population. J Hum Genet 53: 508-514.

6. Park JY, Park SH, Choi JE, Lee SY, Jeon HS et al. (2002) Polymorphisms of the DNA repair gene xeroderma pigmentosum group A and risk of primary lung cancer. Cancer Epidemiol Biomarkers Prev 11: 993-997.

7. Zhang Y, Shu XO, Gao YT, Ji BT, Yang G et al. (2007) Family history of cancer and risk of lung cancer among nonsmoking Chinese women. Cancer Epidemiol Biomarkers Prev 16: 2432-2435.

8. Zheng W, Chow WH, Yang G, Jin F, Rothman N et al. (2005) The Shanghai Women's Health Study: rationale, study design, and baseline characteristics. Am J Epidemiol 162: 1123-1131.

9. Jin G, Xu L, Shu Y, Tian T, Liang J et al. (2009) Common genetic variants on 5p15.33 contribute to risk of lung adenocarcinoma in a Chinese population. Carcinogenesis 30: 987-990.

10. Tang L, Lim W, Eng P, Leong SS, Lim TK et al. (2010) Lung Cancer in Chinese Women: Evidence for an Interaction between Tobacco Smoking and Exposure to Inhalants in the Indoor Environment. Environ Health Perspect. Environ Health Perspect In press.

**Figure Legends**

**Figure S1. Risk of lung cancer associated with rs2736100 for never-smoking female adenocarcinoma cases and never-smoking female controls from East Asia.** GELAC: Genetic Epidemiological Study of Lung Adenocarcinoma (in Taiwan); CAMSCH: Chinese Academy of Medical Sciences Cancer Hospital Study; SNU: Seoul National University study; SWHS: Shanghai Women’s Health Cohort Study; WHLCS: Wuhan Lung Cancer Study; KNUH: Kyungpook National University Study; KUMC: Korea University Study; GEL-S: the Genes and Environment in Lung Cancer, Singapore study; NJLCS: Nanjing Lung Cancer Study. Data from GEL-S were included in Truong et al., (2010) J Natl Cancer Inst, In press and data from NJLCS were included in Jin et al., (2009) Carcinogenesis;30:987-990.

**Figure S2.** **The genotyping cluster plot** **of rs2736100 from (a) Illumina 610K (b) Illumina 370K based on Beadstudio Genotyping Module v3.** The adjusted intensities for each allele are plotted, where each color represents a different genotype in the cluster plots. The genotype call at this locus was confirmed with TaqMan genotyping (concordance of 99.7%).
